# Addressing bias in the definition of SARS-CoV-2 reinfection: implications for underestimation

**DOI:** 10.3389/fmed.2024.1363045

**Published:** 2024-03-11

**Authors:** Hiam Chemaitelly, Houssein H. Ayoub, Patrick Tang, Hadi M. Yassine, Asmaa A. Al Thani, Mohammad R. Hasan, Peter Coyle, Zaina Al-Kanaani, Einas Al-Kuwari, Andrew Jeremijenko, Anvar Hassan Kaleeckal, Ali Nizar Latif, Riyazuddin Mohammad Shaik, Hanan F. Abdul-Rahim, Gheyath K. Nasrallah, Mohamed Ghaith Al-Kuwari, Adeel A. Butt, Hamad Eid Al-Romaihi, Mohamed H. Al-Thani, Abdullatif Al-Khal, Roberto Bertollini, Laith J. Abu-Raddad

**Affiliations:** ^1^Infectious Disease Epidemiology Group, Weill Cornell Medicine-Qatar, Cornell University, Doha, Qatar; ^2^World Health Organization Collaborating Centre for Disease Epidemiology Analytics on HIV/AIDS, Sexually Transmitted Infections, and Viral Hepatitis, Weill Cornell Medicine–Qatar, Cornell University, Doha, Qatar; ^3^Department of Population Health Sciences, Weill Cornell Medicine, Cornell University, New York, NY, United States; ^4^Mathematics Program, Department of Mathematics, Statistics, and Physics, College of Arts and Sciences, Qatar University, Doha, Qatar; ^5^Department of Pathology, Sidra Medicine, Doha, Qatar; ^6^Biomedical Research Center, QU Health, Qatar University, Doha, Qatar; ^7^Department of Biomedical Science, College of Health Sciences, QU Health, Qatar University, Doha, Qatar; ^8^Department of Pathology and Molecular Medicine, McMaster University, Hamilton, ON, Canada; ^9^Hamad Medical Corporation, Doha, Qatar; ^10^Wellcome-Wolfson Institute for Experimental Medicine, Queens University, Belfast, United Kingdom; ^11^Department of Public Health, College of Health Sciences, QU Health, Qatar University, Doha, Qatar; ^12^Primary Health Care Corporation, Doha, Qatar; ^13^Department of Medicine, Weill Cornell Medicine, Cornell University, New York, NY, United States; ^14^Ministry of Public Health, Doha, Qatar; ^15^College of Health and Life Sciences, Hamad bin Khalifa University, Doha, Qatar

**Keywords:** reinfection, bias, time window, immunity, COVID-19, epidemiology

## Abstract

**Introduction:**

Reinfections are increasingly becoming a feature in the epidemiology of severe acute respiratory syndrome coronavirus 2 (SARS-CoV-2) infection. However, accurately defining reinfection poses methodological challenges. Conventionally, reinfection is defined as a positive test occurring at least 90 days after a previous infection diagnosis. Yet, this extended time window may lead to an underestimation of reinfection occurrences. This study investigated the prospect of adopting an alternative, shorter time window for defining reinfection.

**Methods:**

A longitudinal study was conducted to assess the incidence of reinfections in the total population of Qatar, from February 28, 2020 to November 20, 2023. The assessment considered a range of time windows for defining reinfection, spanning from 1 day to 180 days. Subgroup analyses comparing first versus repeat reinfections and a sensitivity analysis, focusing exclusively on individuals who underwent frequent testing, were performed.

**Results:**

The relationship between the number of reinfections in the population and the duration of the time window used to define reinfection revealed two distinct dynamical domains. Within the initial 15 days post-infection diagnosis, almost all positive tests for SARS-CoV-2 were attributed to the original infection. However, surpassing the 30-day post-infection threshold, nearly all positive tests were attributed to reinfections. A 40-day time window emerged as a sufficiently conservative definition for reinfection. By setting the time window at 40 days, the estimated number of reinfections in the population increased from 84,565 to 88,384, compared to the 90-day time window. The maximum observed reinfections were 6 and 4 for the 40-day and 90-day time windows, respectively. The 40-day time window was appropriate for defining reinfection, irrespective of whether it was the first, second, third, or fourth occurrence. The sensitivity analysis, confined to high testers exclusively, replicated similar patterns and results.

**Discussion:**

A 40-day time window is optimal for defining reinfection, providing an informed alternative to the conventional 90-day time window. Reinfections are prevalent, with some individuals experiencing multiple instances since the onset of the pandemic.

## 1 Introduction

Reinfections with severe acute respiratory syndrome coronavirus 2 (SARS-CoV-2) increased as the protection conferred by natural infection waned over time (1, 2). Importantly, this increase was amplified by the emergence of the immune-evasive omicron variant and its subvariants (1–4). The occurrence of reinfections is becoming a regular feature in the epidemiology of SARS-CoV-2, resembling patterns observed in other respiratory infections such as common-cold coronaviruses (5, 6) and influenza (7–10). Gaining insight into the epidemiology of SARS-CoV-2 reinfections is an essential prerequisite for understanding the broader landscape of SARS-CoV-2 epidemiology.

However, defining a SARS-CoV-2 reinfection presents methodological challenges. The most suitable definition, in theory, entails genome sequencing of the virus in every SARS-CoV-2-positive test and evaluating whether the identified virus in a given positive test differs from that detected in the previous positive test (11–13). Implementing this approach is resource-intensive and impractical, especially at this stage of the pandemic.

A pragmatic methodological approach to defining a SARS-CoV-2 reinfection involves applying a time window, allowing for the clearance of an earlier infection to classify a new positive test as a reinfection. Consequently, reinfection is commonly defined as a SARS-CoV-2-positive test occurring at least 90 days after a previous SARS-CoV-2-positive test (3, 14, 15). Despite the fact that the vast majority of SARS-CoV-2 infections resolve within a few days (16, 17), the adoption of this 90-day time window aimed to prevent the misclassification of prolonged infections as reinfections (3, 14, 15), recognizing the persistence of some infections for weeks or even months, albeit rarely (18–20). This choice also accounted for the situation earlier in the pandemic when reinfections were rare (11, 13, 21), emphasizing the importance of distinguishing between two rare events: reinfection versus prolonged infection.

While this definition offers a practical alternative for defining reinfection, it underestimates the occurrence of reinfections, as any true reinfection within 90 days of an earlier infection is not classified as such. The inherent bias in this definition compounds over time, given that this 90-day time window is applied to every subsequent reinfection, precisely when repeat reinfections are becoming increasingly common (22, 23). SARS-CoV-2 waves have been occurring within only a few months of each other, or even occasionally within weeks (24). Therefore, a 90-day threshold may miss many true reinfections in consecutive waves if the time difference between waves is less or comparable to this set 90-day time window.

With the continual evolution of this virus and the emergence of more immune-evasive subvariants (25), this conventional 90-day time window may introduce serious bias in studies of reinfections, potentially leading to incorrect inferences drawn from studies with inaccurately estimated occurrences of reinfections. Importantly, the caution needed to distinguish the rare events of reinfection from prolonged infection early in the pandemic is no longer warranted, as reinfections are no longer rare, while prolonged infections remain as rare as they were before.

To address this challenge, this study explored the possibility of implementing an alternative, shorter time window for defining reinfection. The investigation aims to enhance the methodologies used in studying reinfections and the immune protection of natural infection while mitigating the inherent bias present in the current definition of a 90-day time window.

## 2 Methods

### 2.1 Study population and data sources

The study was conducted on the population of Qatar from February 28, 2020, the date of the first documented SARS-CoV-2 infection, up to November 20, 2023, the date of the end of the study. The analysis utilized the national, federated databases for coronavirus disease 2019 (COVID-19) laboratory testing, vaccination, hospitalization, and death retrieved from the integrated, nationwide digital health information platform (Supplementary Section S1). The platform has captured all SARS-CoV-2-related data with no missing information since the onset of the pandemic, including all polymerase chain reaction (PCR) tests and medically supervised rapid antigen tests (Supplementary Section S2).

All SARS-CoV-2 testing in any facility in Qatar is tracked nationally in one database, the national testing database. This database covers all testing in all locations and facilities throughout the country, whether public or private. SARS-CoV-2 tests are classified on the basis of symptoms and the reason for testing (clinical symptoms, contact tracing, surveys or random testing campaigns, individual requests, routine healthcare testing, pre-travel, at port of entry, or other). Testing is offered free of charge or at heavily subsidized costs depending on the reason for testing within Qatar’s public healthcare system, accessible to all residents irrespective of nationality. These services are available at healthcare centers distributed across the country, catering to the diverse demographic and socio-economic segments of the population.

Up until November 1, 2022, nearly 5% of the population underwent SARS-CoV-2 testing each week, primarily for routine and non-clinical purposes (26). Based on the distribution of the reason for testing up to October 30, 2022, most of the tests in Qatar were conducted for routine reasons, such as being travel-related, and about 75% of documented infections were diagnosed not because of appearance of symptoms, but because of routine testing (26, 27). However, starting from November 1, 2022, the testing rate was reduced to less than 1% per week (28).

The first omicron wave, reaching its peak in January 2022, was of very large magnitude and placed substantial strain on the country’s testing capacity (3, 27, 29). Consequently, rapid antigen testing was introduced and implemented as a substitute for PCR testing, employing identical testing protocols.

The extensive testing approach in Qatar enabled the tracking of reinfections, irrespective of symptomatic presentation, facilitating an opportunity to investigate potential biases in defining reinfection cases.

Qatar initiated its COVID-19 vaccination program in December 2020, utilizing mRNA vaccines and prioritizing individuals based on coexisting conditions and age criteria (26, 30). The vaccination is administered free of charge to all residents, irrespective of nationality, and is centrally tracked at a national level (26, 30).

Qatar has young, diverse demographics; only 9% of its residents are aged 50 or above, with 89% being expatriates from over 150 countries (31). Migrant craft and manual workers constitute about 60% of the population (32, 33), mainly single men aged 20 to 49, hailing predominantly from countries like Bangladesh, India, and Nepal, and working in development projects (34). Consequently, nationality, age, and sex serve as proxies for socio-economic status in this context (31, 33, 35, 36). Further descriptions of Qatar’s population and the national databases have been reported previously (26, 27, 29, 31, 34, 37, 38).

### 2.2 Study design

A longitudinal study was undertaken to assess the incidence of reinfections within the population of Qatar, considering varying time windows for defining reinfection, ranging from 1 day to 180 days. The study cohort encompassed all individuals with a documented SARS-CoV-2-positive test during the study period. In this cohort, which comprised 935,192 individuals, a total of 6,170,451 tests (positive or negative) were conducted from the onset of the pandemic until the conclusion of the study on November 20, 2023. The average testing rate stood at 6.6 tests per person.

Primary infection was defined as the first documented instance of a SARS-CoV-2-positive test for an individual. Reinfection was defined as the first documented SARS-CoV-2-positive test occurring after the completion of the time window used to define reinfection, starting from the last previous SARS-CoV-2 infection diagnosis. The primary outcomes of the study included the total number of documented reinfections in the population and the maximum number of observed reinfections experienced by any given individual in the population, both examined across the various time window definitions investigated in this study.

In essence, the concept of the present study is that the relationship between the total number of reinfections in the population and the duration of the time window used to define reinfection may reveal clearly distinct dynamical domains, enabling an informed decision on setting the time window to define reinfection. The existence of two distinct dynamical domains is a reflection of the existence of two different population distributions influencing this relationship. The first is the distribution of clearing the infection, and the second is the distribution of the incidence of reinfection.

### 2.3 Statistical analysis

Frequency distributions and measures of central tendency were employed to characterize measures within the study cohort. Statistical analyses calculated the total number of documented reinfections in the population and the maximum number of observed reinfections experienced by any given individual, considering varying time windows for defining reinfection, ranging from 1 day to 180 days. The total number of documented first, second, third, and fourth reinfections in the population were also computed for the different time windows. This latter investigation aimed to assess whether distinct time window definitions are warranted for repeat reinfections compared to the first reinfection.

Documented reinfections constitute only a subset of all potential reinfections in a population, as many may go undocumented through a SARS-CoV-2 test. Patterns for undocumented infections may deviate from those that are documented. To address this, the study analyses were repeated in a sensitivity analysis including only high testers in the study cohort, a subset of the population less impacted by undocumented infections due to frequent repeat testing, often for routine reasons such as employment or travel (26, 27).

High testers were defined as individuals in the top 10th percentile of the real-world testing frequency distribution, encompassing all reasons for testing. According to this distribution, high testers are individuals with a testing rate of ≥3.4 tests per person-year. The consistency of patterns and results among high testers with those in the full cohort would support the conclusion that the proposed time window in this study may not have been influenced by the occurrence of undocumented infections. Statistical analyses were performed using Stata/SE version 18.0 (Stata Corporation, College Station, TX, USA).

A second sensitivity analysis was undertaken to examine the consistency of reinfection patterns observed in the main analysis, encompassing all times during the pandemic, when the analysis is restricted to the four largest SARS-CoV-2 infection waves, each dominated by a distinct variant (4, 21, 31, 39).

### 2.4 Oversight

The institutional review boards at Hamad Medical Corporation and Weill Cornell Medicine–Qatar approved this retrospective study with a waiver of informed consent. The study was reported according to the Strengthening the Reporting of Observational Studies in Epidemiology (STROBE) guidelines (Supplementary Table S1).

## 3 Results

### 3.1 Optimizing the time window for defining reinfection

Table 1 shows the characteristics of the study cohort, comprising all individuals in Qatar with a documented SARS-CoV-2 infection during the study period. The study population is representative of the internationally diverse, yet predominantly young and male demographic of the country.

**Table 1:** Baseline characteristics of the study population.

Characteristics	Study cohort *N* (%)
Total *N*	935,192
Median age (IQR)—years	33.0 (24.0–41.0)
Age—years	
0–9 years	90,028 (9.6)
10–19 years	87,116 (9.3)
20–29 years	192,565 (20.6)
30–39 years	296,309 (31.7)
40–49 years	167,292 (17.9)
50–59 years	70,175 (7.5)
60–69 years	23,214 (2.5)
≥70 years	8,493 (0.9)
Sex	
Male	584,687 (62.5)
Female	350,505 (37.5)
Nationality^*^	
Bangladeshi	52,762 (5.6)
Egyptian	48,945 (5.2)
Filipino	93,921 (10.0)
Indian	210,315 (22.5)
Nepalese	65,128 (7.0)
Pakistani	39,568 (4.2)
Qatari	176,951 (18.9)
Sri Lankan	24,255 (2.6)
Sudanese	24,085 (2.6)
Other nationalities^†^	199,262 (21.3)
Number of coexisting conditions	
None	724,513 (77.5)
1	115,788 (12.4)
2	48,373 (5.2)
3	20,639 (2.2)
4	11,533 (1.2)
5	6,918 (0.7)
≥6	7,428 (0.8)
Vaccination^‡^	
Unvaccinated	564,670 (60.4)
1 dose	19,657 (2.1)
2 doses	270,227 (28.9)
3 doses	79,666 (8.5)
≥4 doses	972 (0.1)

IQR, interquartile range; SARS-CoV-2, severe acute respiratory syndrome coronavirus 2.

^*^Nationalities were chosen to represent the most populous groups in Qatar.

^†^These comprise 173 other nationalities.

^‡^Ascertained at time of primary infection.

**Figure 1:**
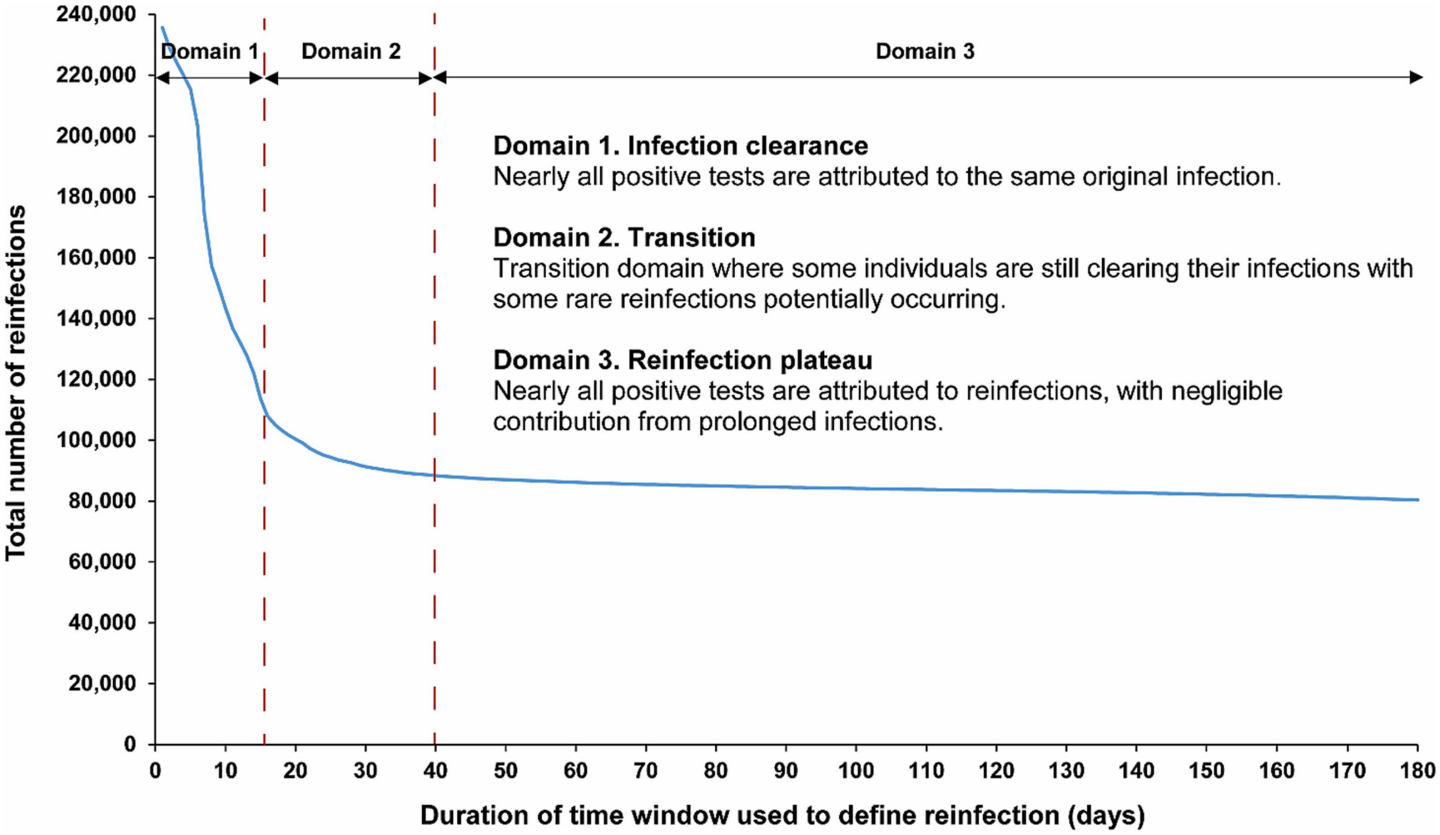
Total number of reinfections in the population versus the time-window duration used for defining reinfection. The dashed line at day 40 represents the proposed optimal time window for defining a reinfection.

Figure 1 illustrates the total estimated number of reinfections in the population versus the time-window duration used for defining reinfection. Initially, the total number of reinfections declines rapidly, from 235,660 when the time window is set at only 1 day to 113,649 when the time window is extended to 15 days after the previous infection diagnosis. This swift decline supports the notion that many individuals are testing positive within the first 15 days of a positive test. At the same time, it also indicates that more and more individuals are clearing the infection, leading to a progressive increase in the number of people testing negative with each passing day within this 15-day duration. This trend is consistent with the vast majority of infected persons clearing their infection within 15 days of diagnosis.

Following this initial rapid decline, a pronounced shift in the curve’s shape becomes evident within the time window spanning from 16 days to 30 days (Figure 1). This transition to a new shape of the curve indicates the presence of two distinct dynamical domains: one where nearly all positive tests are attributable to the same original infection before day 15 after the previous infection diagnosis, and another, following the transition, where nearly all positive tests after day 30 are attributed to reinfections, with negligible contribution from prolonged infections. These two domains are labeled thereafter as the “infection clearance” and “reinfection plateau” domains, respectively.

These two clearly distinct domains emerge because two different population distributions dominate the relationship between the total number of reinfections and the duration of the time window at *different* times post-infection (Figure 1). The distribution of clearing the infection predominates in the infection clearance domain, while the distribution of the incidence of reinfection dominates in the reinfection plateau domain. The transition between these domains, occurring from 16 days to 30 days post-infection, is influenced by both of these distributions.

Given the existence of these two clearly distinct dynamics, choosing a time window for defining reinfection at 40 days strikes a balance. It is adequately conservative in defining a reinfection (as opposed to a prolonged infection) while not missing many reinfections compared to a longer time window. Setting the time window at 40 days, instead of the conventional 90-day window, increases the total estimated number of reinfections in the population from 84,565 to 88,384 reinfections, capturing an additional 4.3% of reinfections that would have been missed by applying the conventional 90-day time window.

### 3.2 Time window for first and repeat reinfections

**Figure 2:**
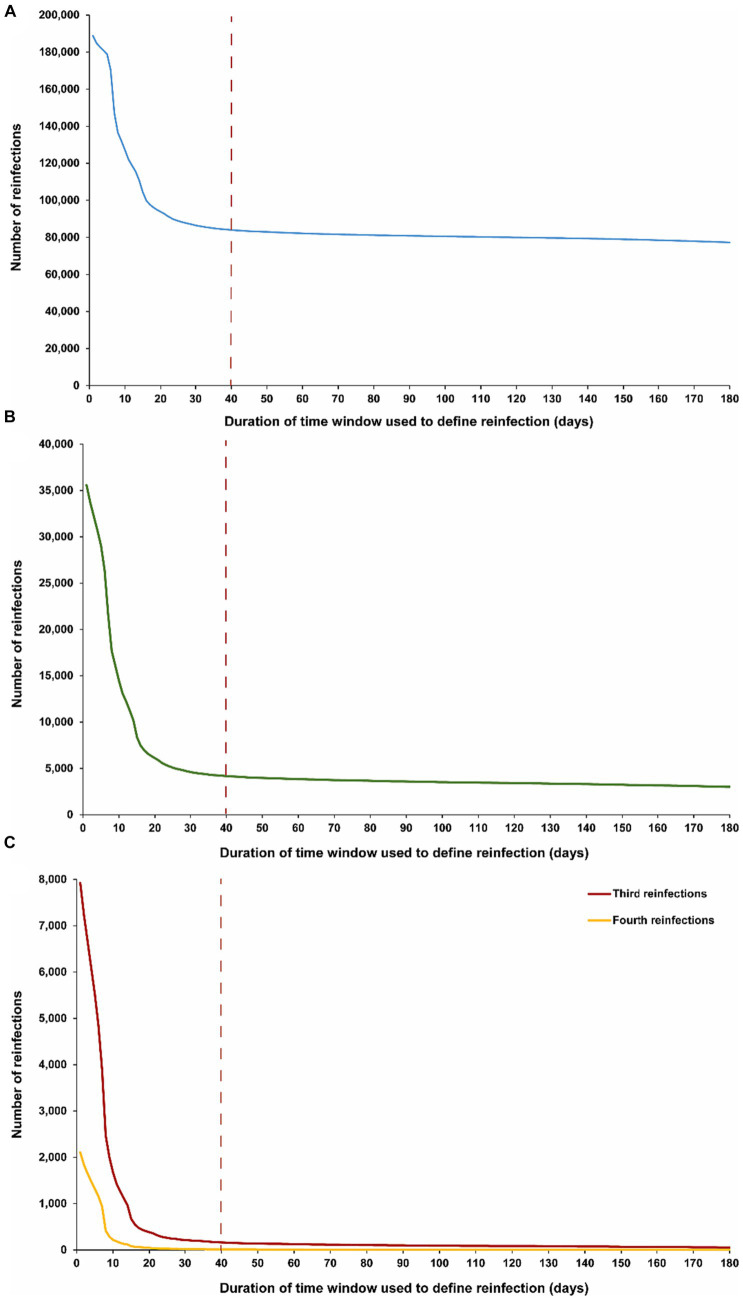
Number of **(A)** first reinfections, **(B)** second reinfections and **(C)** third, and fourth reinfections in the population versus the time-window duration used for defining reinfection. The dashed line represents the proposed optimal time window for defining a reinfection.

The above analysis indicates an optimal choice of a 40-day time window for defining reinfection. Figure 2 explores the applicability of this time window individually for each of the first, second, third, and fourth reinfections. The results demonstrate that a time window of 40 days is appropriate for defining reinfection, regardless of whether it is a first, second, third, or fourth occurrence. The number of reinfections versus the time-window duration followed a largely consistent pattern, irrespective of whether the reinfection was a first reinfection or a repeat reinfection.

Notably, in the analyses of third and fourth reinfections, the transition in the shape of the number of reinfections versus the time-window duration appears to occur more rapidly, reaching the reinfection plateau sooner. This supports the possibility of an even shorter time window than 40 days for defining reinfection. This may be attributed to the fact that, by the time individuals in this population experienced their third or fourth reinfections during the pandemic, there was limited testing in the initial days after the infection to assess clearance, unlike in earlier stages. It could also be a result of faster clearance of reinfections, especially repeat occurrences (40–42).

### 3.3 Maximum number of reinfections in the population

**Figure 3:**
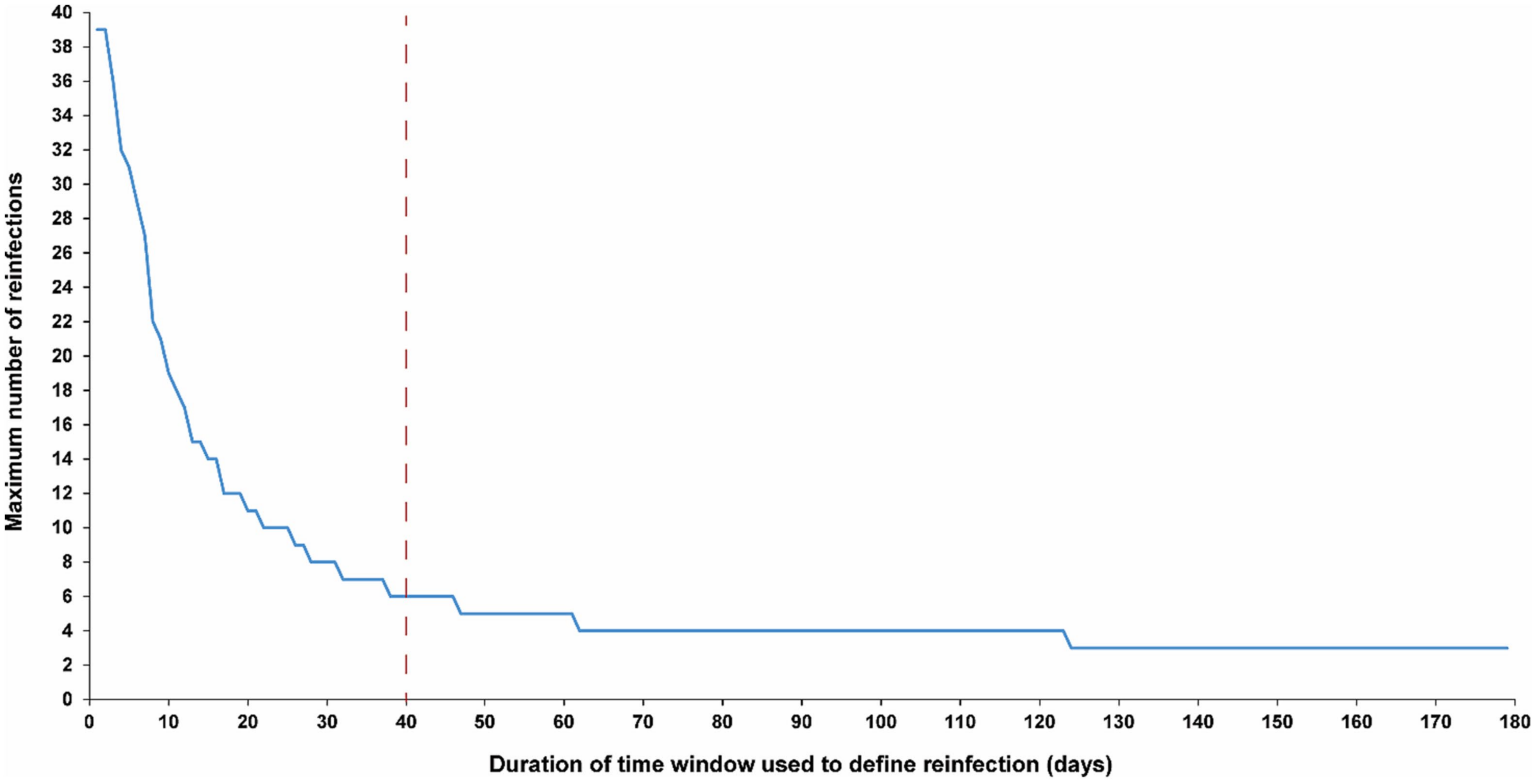
Maximum number of observed reinfections experienced by any given individual in the population versus the time-window duration used for defining reinfection. The dashed line represents the proposed optimal time window for defining a reinfection.

Figure 3 illustrates the maximum number of observed reinfections experienced by any given individual in the population versus the time-window duration used for defining reinfection. This figure highlights the relevance of an appropriate definition for the time window in capturing the phenomenon of repeat reinfections. For example, if the time window is set at 15 days, at least one individual in the population would have been estimated to have experienced 14 reinfections throughout the pandemic. Meanwhile, the maximum number of observed reinfections is 8, 6, and 4 if the time window was set at 30, 40, and 90 days, respectively.

### 3.4 Sensitivity analysis: results for only high testers

**Figure 4:**
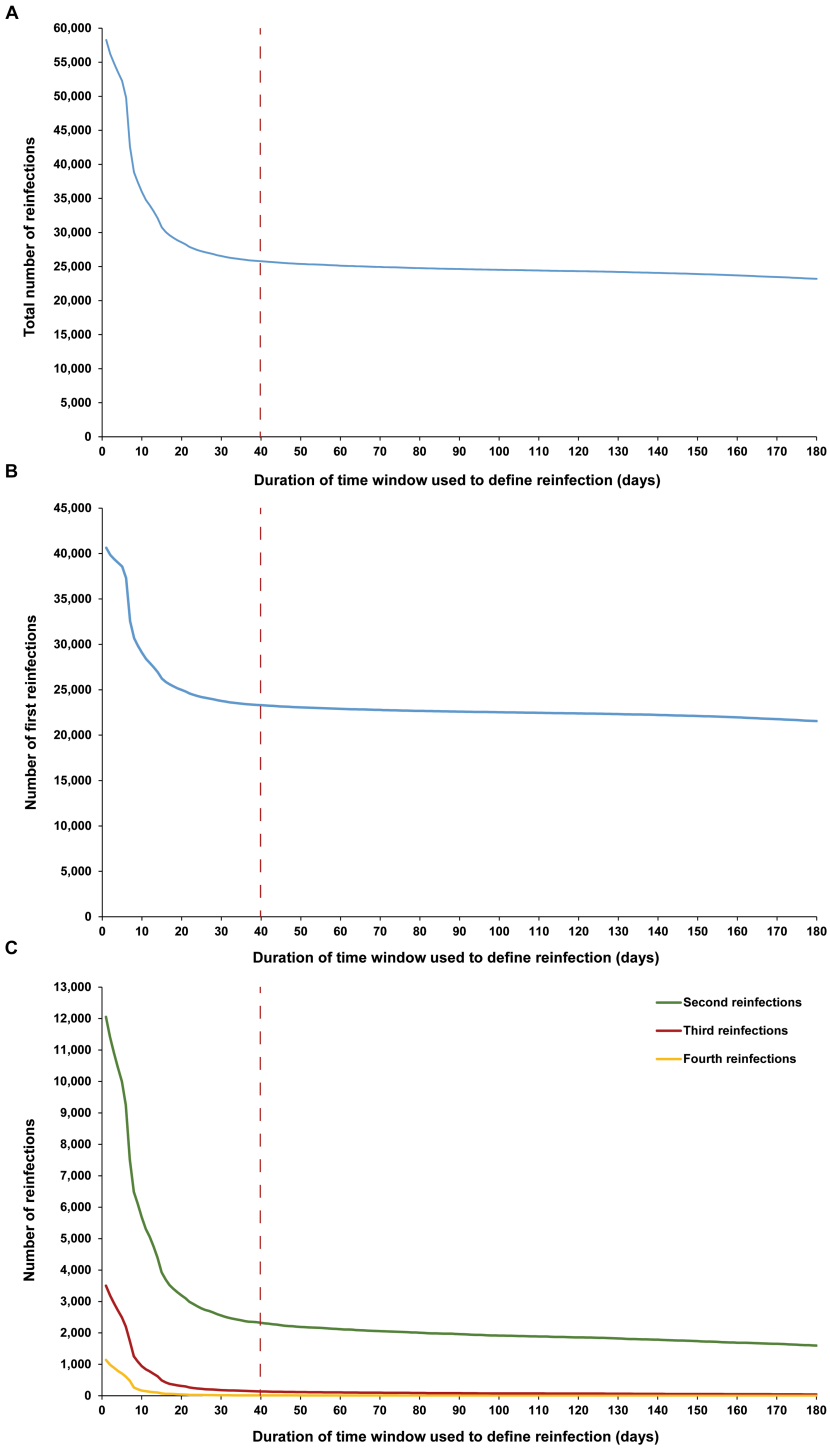
Number of **(A)** all reinfections, **(B)** first reinfections, and **(C)** second, third, and fourth reinfections among high testers in the population versus the time-window duration used for defining reinfection. High testers were defined as individuals in the top 10th percentile of the testing frequency distribution. The dashed line represents the proposed optimal time window for defining a reinfection.

**Figure 5:**
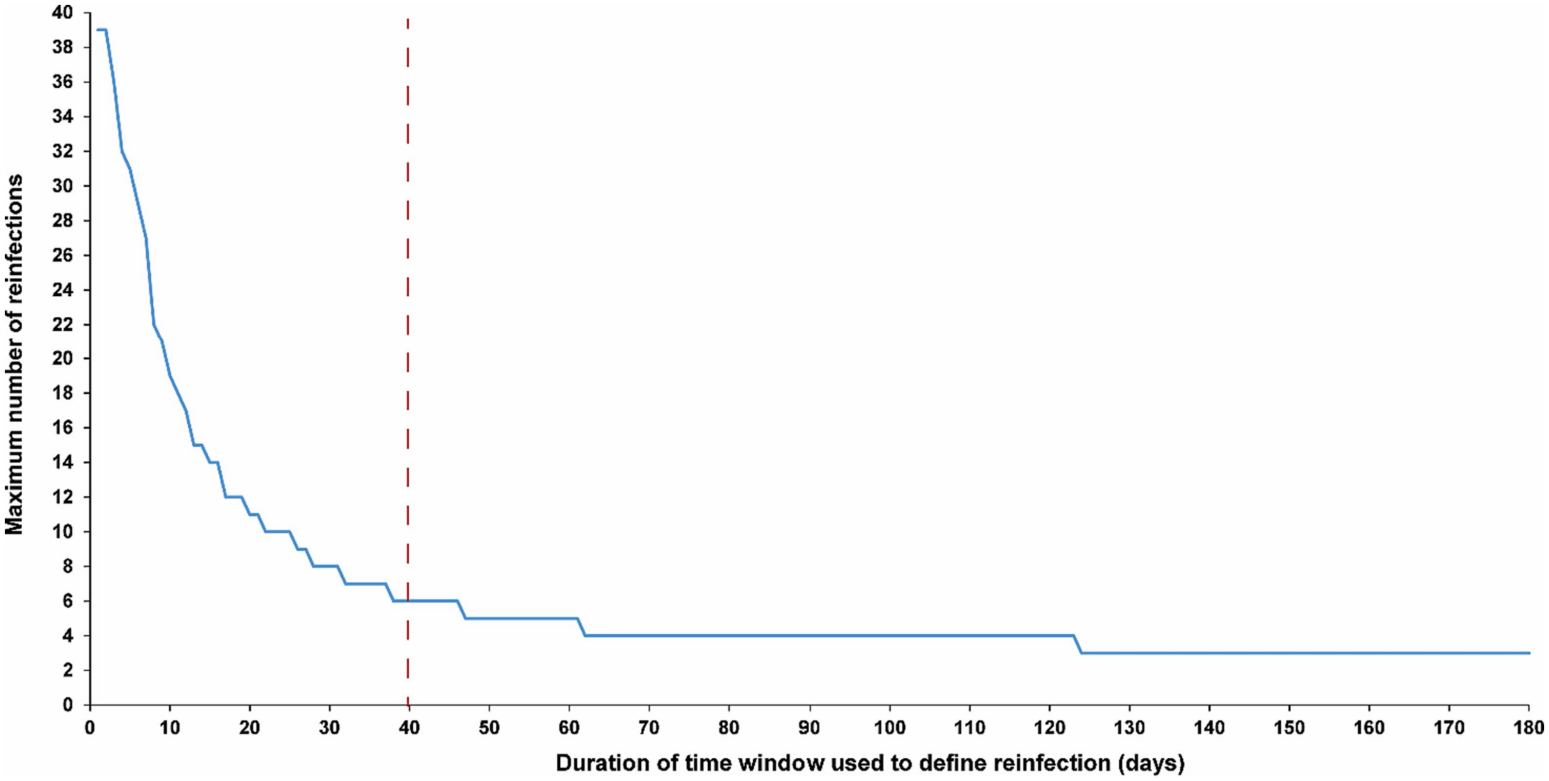
Maximum number of observed reinfections experienced by any given individual among high testers in the population versus the time-window duration used for defining reinfection. High testers were defined as individuals in the top 10th percentile of the testing frequency distribution. The dashed line represents the proposed optimal time window for defining a reinfection.

The sensitivity analysis, restricted to only high testers in the population, reproduced the same patterns and results as those observed for the entire population. This held true for all study outcomes, including the total number of reinfections (Figure 4A), the number of each of the first, second, third, and fourth reinfections (Figures 4B,C), and the maximum number of observed reinfections (Figure 5). The analysis affirmed the 40-day time window as an optimal choice, suggesting that the conclusions drawn above regarding setting the time window are unlikely to have been altered by the occurrence of infections that were never documented.

### 3.5 Sensitivity analysis: reinfection patterns in distinct waves

**Figure 6:**
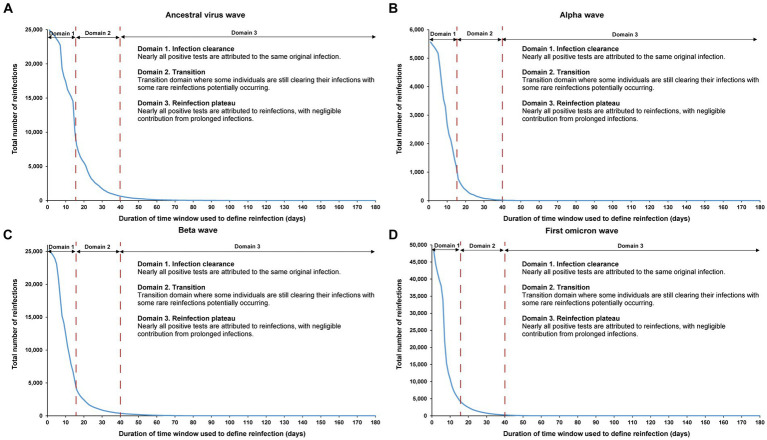
Reinfection patterns in distinct waves. Total number of reinfections in the population versus the time-window duration used for defining reinfection during the **(A)** ancestral virus wave (February 28, 2020-July 31, 2020), **(B)** alpha wave (January 18, 2021-March 7, 2021), **(C)** beta wave (March 8, 2021-May 31, 2021), and **(D)** first omicron wave (December 19, 2021-February 28, 2022). The dashed line at day 40 represents the proposed optimal time window for defining a reinfection. All curves converge to zero at large durations of the time window due to the relatively short duration of each wave in comparison to the total study duration.

The sensitivity analysis, restricting the analysis to each of the four largest SARS-CoV-2 infection waves, each dominated by a distinct variant, showed the same reinfection patterns observed in the main analysis encompassing all times during the pandemic (Figure 6). This analysis affirmed the 40-day time window as an optimal choice, independent of the wave’s size or the variant that dominated the wave.

The analysis also indicated an increasingly steeper slope in viral clearance over time, especially during the first omicron wave. This trend may have been due to progressive changes in retesting requirements for individuals in isolation following documented infection, as well as the introduction of rapid antigen testing during the first omicron wave.

## 4 Discussion

Investigating the empirical dependence of the estimated number of reinfections in the population on the time-window duration used for defining reinfection has revealed the existence of two distinct dynamical domains, providing insights for a more effective definition of reinfection that is less susceptible to potential bias. In the first 15 days after an infection is diagnosed, labeled here as the infection clearance domain, nearly all SARS-CoV-2 positive tests are attributable to the same original infection. However, beyond the 30-day mark post-infection, within the reinfection plateau domain, nearly all positive tests are attributed to reinfections, with a negligible contribution from prolonged infections. These findings underscore that a time window of 40 days serves as an adequately conservative definition for reinfection, superseding the current conventional definition of a 90-day time window.

This conclusion emphasizes that the conventional 90-day time window is overly restrictive, resulting in significant bias in capturing reinfections and potentially leading to inaccurate or imprecise estimates in studies of reinfections, including those examining the immune protection of natural infection against reinfection. This limitation is particularly critical in the current stage of the pandemic when reinfections are common, and accurately capturing repeat reinfections is essential for a meaningful understanding of the current epidemiology of SARS-CoV-2 infection.

These findings enable the estimation of protection of natural immunity within the time window spanning from 40 to 90 days after a first infection, which is not possible under the conventional definition. Moreover, they indicate that studies assessing the protective effects of natural infection against reinfection might have overestimated this protection, especially when relying on short follow-up periods after the initial infection. It is recommended that future studies present results using both the conventional 90-day window and the proposed 40-day window to assess potential biases and elucidate the implications of adopting the new proposed time window.

An important finding from this study is the higher incidence of reinfections compared to common perception. The 90-day time window missed a proportion of reinfections relative to the 40-day time window. Instances were identified where individuals experienced up to 6 documented reinfections over the nearly 4 years of the pandemic. Given that documented reinfections represent only a fraction of the total, which includes also undocumented reinfections, this implies that reinfections are substantially underestimated. This finding suggests a resemblance in reinfection patterns across various respiratory infections, encompassing SARS-CoV-2, common-cold coronaviruses (5, 6), and influenza (7–10). Furthermore, it aligns with experimental observations derived from sequential influenza challenge studies (10). This underscores the imperative for enhanced understanding of the epidemiology of reinfections to unravel the factors contributing to the “leaky” immune protection enabling their occurrence. The incidence of reinfections increases the risk of virus mutation and evolution due to increased transmissions in the population.

This study has limitations. The definition of reinfection was deduced through the observation of infection patterns, departing from the conventional methodology of genome sequencing for every SARS-CoV-2-positive test (11–13). The latter approach entails evaluating whether the identified virus in a specific positive test differs from that detected in the preceding positive test (11–13). However, the practical implementation of the conventional approach, especially in the current stage of the pandemic, is not feasible. The outcomes of the application of the conventional approach during the early stages of the pandemic are, on the whole, consistent with the findings of the present analysis (11, 13). Notably, the conventional method has also proven to be intricate and often inconclusive when distinguishing reinfections from prolonged infections (11, 13). This complexity is exemplified in cases where only a few changes in allele frequency are observed (11, 13).

In lieu of the conventional method, we presented a novel approach, which, to the best of our knowledge, has not been previously utilized for either SARS-CoV-2 infection or any other infection. The conceptual foundation of this approach stems from recognizing two specific population distributions behind the relationship between the estimated number of reinfections and the duration of the time window used for defining reinfection. The first distribution pertains to the clearance of the infection, while the second relates to the incidence of reinfection. The observation of two discernible dynamical domains, along with a transition region between them, strongly implies that the clearance of infection dominates the first domain, while the distribution of reinfection incidence dominates the second domain.

The present analysis was conducted on the population of Qatar, characterized by a predominantly young demographic composition. Consequently, the findings may lack generalizability to other countries where elderly citizens constitute a more substantial proportion of the population. The reliance on documented infections may introduce bias, as patterns for undocumented infections may differ from those documented. Furthermore, variations in testing frequency across different population segments and over time can lead to fluctuations in the likelihood of documenting infections. High testers may not be representative of the broader population due to self-selection influenced by factors such as perceived risk associated with occupation, living conditions, vaccination status, or underlying health conditions.

However, a strength of this study lies in its comprehensive scope, encompassing the entire population of a country with high testing rates. This approach enhances the capture of infections and reinfections, contributing to the robustness of the study’s findings. Additionally, the sensitivity analysis, focusing exclusively on high testers less impacted by undocumented infections, generated similar results, suggesting that the study findings are less likely to be influenced by undocumented infections.

## 5 Conclusion

A 40-day time window serves as an appropriately conservative definition for reinfection, offering an informed alternative to the current conventional 90-day time window. The latter, shown to be unnecessarily restrictive, introduces bias in reinfection capture that may jeopardize estimates in reinfection studies. Contrary to common perception, reinfections are more prevalent, with some individuals experiencing multiple instances since the onset of the pandemic. A nuanced understanding of the factors contributing to the “leaky” immune protection allowing for this heightened incidence of reinfections is warranted.

## Data availability statement

The original contributions presented in the study are included in the article/Supplementary materials, further inquiries can be directed to the corresponding authors.

## Ethics statement

The studies involving humans were approved by Hamad Medical Corporation and Weill Cornell Medicine–Qatar Institutional Review Boards. The studies were conducted in accordance with the local legislation and institutional requirements. Written informed consent for participation was not required from the participants or the participants’ legal guardians/next of kin in accordance with the national legislation and institutional requirements.

## Author contributions

HC: Conceptualization, Data curation, Formal analysis, Investigation, Methodology, Writing – original draft, Writing – review & editing. HA: Data curation, Writing – review & editing. PT: Data curation, Writing – review & editing. HMY: Data curation, Writing – review & editing. AAAT: Data curation, Writing – review & editing. MRH: Data curation, Writing – review & editing. PC: Data curation, Writing – review & editing. ZA-K: Data curation, Writing – review & editing. EA-K: Data curation, Writing – review & editing. AJ: Data curation, Writing – review & editing. AHK: Data curation, Writing – review & editing. ANL: Data curation, Writing – review & editing. RS: Data curation, Writing – review & editing. HFA-R: Data curation, Writing – review & editing. GKN: Data curation, Writing – review & editing. MGA-K: Data curation, Writing – review & editing. AAB: Data curation, Writing – review & editing. HEA-R: Data curation, Writing – review & editing. MHA-T: Data curation, Writing – review & editing. AA-K: Data curation, Writing – review & editing. RB: Data curation, Writing – review & editing. LJA-R: Conceptualization, Data curation, Funding acquisition, Investigation, Methodology, Project administration, Supervision, Writing – original draft, Writing – review & editing.
