# Supplementary material for: Addressing bias in the definition of SARS-CoV-2 reinfection: implications for underestimation
Source: Front Med (Lausanne). 2024 Mar 11;11:1363045. doi: 10.3389/fmed.2024.1363045 (PMC10961414; doi:10.3389/fmed.2024.1363045)
Supplement: Supplementary file 1 [file Data_Sheet_1.PDF]

Supplementary Material

Table of Contents

1. Supplementary Section S1. Study population and data sources 2
2. Supplementary Section S2. Laboratory methods and variant ascertainment 4
   Real-time reverse-transcription polymerase chain reaction testing 4
   Rapid antigen testing 4
   Classification of infections by variant type 4
Supplementary Table S1. STROBE checklist for cohort studies. 5
References 7

1. Supplementary Section S1. Study population and data sources

This study was conducted on the population of Qatar including data between February 28, 2020, marking the first documented severe acute respiratory syndrome coronavirus 2 (SARS-CoV-2) infection in Qatar, and November 20, 2023, end of the study. It analyzed the national, federated databases for coronavirus disease 2019 (COVID-19) laboratory testing, vaccination, hospitalization, and death, retrieved from the integrated, nationwide, digital-health information platform. Databases include all SARS-CoV-2-related data with no missing information since the onset of the pandemic, including all polymerase chain reaction (PCR) tests and medically supervised rapid antigen tests (Supplementary Section S2).

Qatar's national and universal public healthcare system uses the Cerner-system advanced digital health platform to track all electronic health record encounters of each individual in the country, including all citizens and residents registered in the national and universal public healthcare system. Registration in the public healthcare system is mandatory for citizens and residents.

The databases analyzed in this study are data-extract downloads from the Cerner-system that have been implemented on a regular schedule since the onset of pandemic by the Business Intelligence Unit at Hamad Medical Corporation. Hamad Medical Corporation is the national public healthcare provider in Qatar. At every download all tests, COVID-19 vaccinations, hospitalizations related to COVID-19, and all death records regardless of cause are provided to the authors through .csv files. These databases have been analyzed throughout the pandemic not only for study-related purposes, but also to provide policymakers with summary data and analytics to inform the national response.

Every health encounter in the Cerner-system is linked to a unique individual through the HMC Number that links all records for this individual at the national level. Databases were merged and analyzed using the HMC Number to link all records whether for testing, vaccinations, hospitalizations, and deaths. All deaths in Qatar are tracked by the public healthcare system (1, 2). All COVID-19-related healthcare was provided only in the public healthcare system. No private entity was permitted to provide COVID-19-related hospitalization. COVID-19 vaccination was also provided only through the public healthcare system. These health records were tracked throughout the COVID-19 pandemic using the Cerner system. This system has been implemented in 2013, before the onset of the pandemic. Therefore, we had all health records related to this study for the full national cohort of citizens and residents throughout the pandemic. This allowed us to follow each person over time.

Demographic details for every HMC Number (individual) such as sex, age, and nationality are collected upon issuing of the universal health card, based on the Qatar Identity Card, which is a mandatory requirement by the Ministry of Interior to every citizen and resident in the country. Date of expiry of Qatar Identity Card is collected and updated at encounters with the public healthcare system. Data extraction from the Qatar Identity Card to the digital health platform is performed electronically through scanning techniques.

All SARS-CoV-2 testing in any facility in Qatar is tracked nationally in one database, the national testing database. This database covers all testing in all locations and facilities throughout the country, whether public or private. Every PCR test and a proportion of the facility-based

rapid antigen tests conducted in Qatar, regardless of location or setting, are classified on the basis of symptoms and the reason for testing (clinical symptoms, contact tracing, surveys or random testing campaigns, individual requests, routine healthcare testing, pre-travel, at port of entry, or other).

Before November 1, 2022, SARS-CoV-2 testing in Qatar was done at a mass scale where close to 5% of the population were tested every week (3, 4). Based on the distribution of the reason for testing up to October 30, 2022, most of the tests in Qatar were conducted for routine reasons, such as being travel-related, and about 75% of documented infections were diagnosed not because of appearance of symptoms, but because of routine testing (3, 4).

Starting from November 1, 2022, SARS-CoV-2 testing was substantially reduced, but still close to 1% of the population are tested every week (5). All testing results in the national testing database during the present study were factored in the analyses of this study.

The first large omicron wave that peaked in January of 2022 was massive and strained the testing capacity in the country (3, 6, 7). Accordingly, rapid antigen testing was introduced to relieve the pressure on PCR testing. Implementation of this change in testing occurred quickly precluding incorporation of reason for testing in large proportion of the rapid antigen tests for several months. While the reason for testing is available for all PCR tests, it is not available for all rapid antigen tests. Availability of reason for testing for the rapid antigen tests also varied with time.

Rapid antigen test kits are available for purchase in pharmacies in Qatar, but outcome of home-based testing is not reported nor documented in the national databases. Since SARS-CoV-2-test outcomes are linked to specific public health measures, restrictions, and privileges, testing policy and guidelines stress facility-based testing as the core testing mechanism in the population. While facility-based testing is provided free of charge or at low subsidized costs, depending on the reason for testing, home-based rapid antigen testing is de-emphasized and not supported as part of national policy.

Further descriptions of the study population and these national databases were reported previously (1-4, 7-10).

2. Supplementary Section S2. Laboratory methods and variant ascertainment

Real-time reverse-transcription polymerase chain reaction testing

Nasopharyngeal and/or oropharyngeal swabs were collected for polymerase chain reaction (PCR) testing and placed in Universal Transport Medium (UTM). Aliquots of UTM were: 1) extracted on KingFisher Flex (Thermo Fisher Scientific, USA), MGISP-960 (MGI, China), or ExiPrep 96 Lite (Bioneer, South Korea) followed by testing with real-time reverse-transcription PCR (RT-qPCR) using TaqPath COVID-19 Combo Kits (Thermo Fisher Scientific, USA) on an ABI 7500 FAST (Thermo Fisher Scientific, USA); 2) tested directly on the Cepheid GeneXpert system using the Xpert Xpress SARS-CoV-2 (Cepheid, USA); or 3) loaded directly into a Roche cobas 6800 system and assayed with the cobas SARS-CoV-2 Test (Roche, Switzerland). The first assay targets the viral S, N, and ORF1ab gene regions. The second targets the viral N and E-gene regions, and the third targets the ORF1ab and E-gene regions.

All PCR testing was conducted at the Hamad Medical Corporation Central Laboratory or Sidra Medicine Laboratory, following standardized protocols.

Rapid antigen testing

Severe acute respiratory syndrome coronavirus 2 (SARS-CoV-2) antigen tests were performed on nasopharyngeal swabs using one of the following lateral flow antigen tests: Panbio COVID-19 Ag Rapid Test Device (Abbott, USA); SARS-CoV-2 Rapid Antigen Test (Roche, Switzerland); Standard Q COVID-19 Antigen Test (SD Biosensor, Korea); or CareStart COVID-19 Antigen Test (Access Bio, USA). All antigen tests were performed point-of-care according to each manufacturer's instructions at public or private hospitals and clinics throughout Qatar with prior authorization and training by the Ministry of Public Health (MOPH). Antigen test results were electronically reported to the MOPH in real time using the Antigen Test Management System which is integrated with the national Coronavirus Disease 2019 (COVID-19) database.

Classification of infections by variant type

Surveillance for SARS-CoV-2 variants in Qatar is based on viral genome sequencing and multiplex RT-qPCR variant screening (11) of random positive clinical samples (4, 12-16), complemented by deep sequencing of wastewater samples (14, 17, 18). Further details on the viral genome sequencing and multiplex RT-qPCR variant screening throughout the SARS-CoV-2 waves in Qatar can be found in previous publications (3, 4, 6, 10, 12-16, 19-23).

Supplementary Table S1. STROBE checklist for cohort studies.

	Item No	Recommendation	Main Text page
**Title and abstract**	1	(*a*) Indicate the study's design with a commonly used term in the title or the abstract<br>(*b*) Provide in the abstract an informative and balanced summary of what was done and what was found	Abstract
**Introduction**			
Background/rationale	2	Explain the scientific background and rationale for the investigation being reported	Introduction
Objectives	3	State specific objectives, including any prespecified hypotheses	Introduction
**Methods**			
Study design	4	Present key elements of study design early in the paper	Methods ('Study design')
Setting	5	Describe the setting, locations, and relevant dates, including periods of recruitment, exposure, follow-up, and data collection	Methods ('Study population and data sources' & 'Study design') & Supplementary Section S1
Participants	6	(*a*) Give the eligibility criteria, and the sources and methods of selection of participants. Describe methods of follow-up<br>(*b*) For matched studies, give matching criteria and number of exposed and unexposed	Methods ('Study population and data sources' & 'Study design') & Supplementary Section S1
Variables	7	Clearly define all outcomes, exposures, predictors, potential confounders, and effect modifiers. Give diagnostic criteria, if applicable	Methods ('Study population and data sources', 'Study design' & 'Statistical analysis')
Data sources/ measurement	8*	For each variable of interest, give sources of data and details of methods of assessment (measurement). Describe comparability of assessment methods if there is more than one group	Methods ('Study population and data sources', 'Study design'& 'Statistical analysis') & Supplementary Sections S1-S2
Bias	9	Describe any efforts to address potential sources of bias	Methods ('Statistical analysis')
Study size	10	Explain how the study size was arrived at	Methods ('Study design')
Quantitative variables	11	Explain how quantitative variables were handled in the analyses. If applicable, describe which groupings were chosen and why	Methods ('Study design'& 'Statistical analysis')
Statistical methods	12	(*a*) Describe all statistical methods, including those used to control for confounding	Methods ('Statistical analysis')
		(*b*) Describe any methods used to examine subgroups and interactions	Methods ('Statistical analysis')
		(*c*) Explain how missing data were addressed	Not applicable, see Methods ('Study population and data sources') & Supplementary Section S1
		(*d*) If applicable, explain how loss to follow-up was addressed	Not applicable, see Methods ('Study population and data sources') & Supplementary Section S1
		(*e*) Describe any sensitivity analyses	Methods ('Statistical analysis')
**Results**			
Participants	13*	(a) Report numbers of individuals at each stage of study—eg numbers potentially eligible, examined for eligibility, confirmed eligible, included in the study, completing follow-up, and analysed<br>(b) Give reasons for non-participation at each stage<br>(c) Consider use of a flow diagram	Methods ('Study design'), Results ('Optimizing the time window for defining reinfection'), Table 1 & Figure 1
Descriptive data	14	(a) Give characteristics of study participants (eg demographic, clinical, social) and information on exposures and potential confounders	Table 1
		(b) Indicate number of participants with missing data for each variable of interest	Not applicable, see Methods ('Study population and data sources') & Supplementary Section S1
		(c) Summarise follow-up time (eg, average and total amount)	Not applicable
Outcome data	15	Report numbers of outcome events or summary measures over time	Results ('Optimizing the time window for defining reinfection', 'Time window for first and repeat reinfections', & 'maximum number of reinfections in the population') & Figures 1-3
Main results	16	(a) Give unadjusted estimates and, if applicable, confounder-adjusted estimates and their precision (eg, 95% confidence interval). Make	Results ('Optimizing the time window for defining

		clear which confounders were adjusted for and why they were included	reinfection', 'Time window for first and repeat reinfections', & 'maximum number of reinfections in the population') & Figures 1-3
		(b) Report category boundaries when continuous variables were categorized	Table 1
		(c) If relevant, consider translating estimates of relative risk into absolute risk for a meaningful time period	Not applicable
Other analyses	17	Report other analyses done—eg analyses of subgroups and interactions, and sensitivity analyses	Results ('Sensitivity analysis: Results for only high testers' & 'Sensitivity analysis: Reinfection patterns in distinct waves') & Figures 4-6
Discussion			
Key results	18	Summarise key results with reference to study objectives	Discussion, paragraphs 1-4
Limitations	19	Discuss limitations of the study, taking into account sources of potential bias or imprecision. Discuss both direction and magnitude of any potential bias	Discussion, paragraphs 5 and 7
Interpretation	20	Give a cautious overall interpretation of results considering objectives, limitations, multiplicity of analyses, results from similar studies, and other relevant evidence	Conclusion
Generalisability	21	Discuss the generalisability (external validity) of the study results	Discussion, paragraph 7
Other information			
Funding	22	Give the source of funding and the role of the funders for the present study and, if applicable, for the original study on which the present article is based	Funding

References

1. AlNuaimi AA, Chemaitelly H, Semaan S, AlMukdad S, Al-Kanaani Z, Kaleeckal AH, et al. All-cause and COVID-19 mortality in Qatar during the COVID-19 pandemic. BMJ Glob Health. 2023;8(5).
2. Chemaitelly H, Faust JS, Krumholz HM, Ayoub HH, Tang P, Coyle P, et al. Short- and longer-term all-cause mortality among SARS-CoV-2- infected individuals and the pull-forward phenomenon in Qatar: a national cohort study. Int J Infect Dis. 2023;136:81-90.
3. Altarawneh HN, Chemaitelly H, Ayoub HH, Tang P, Hasan MR, Yassine HM, et al. Effects of Previous Infection and Vaccination on Symptomatic Omicron Infections. N Engl J Med. 2022;387(1):21-34.
4. Chemaitelly H, Tang P, Hasan MR, AlMukdad S, Yassine HM, Benslimane FM, et al. Waning of BNT162b2 Vaccine Protection against SARS-CoV-2 Infection in Qatar. N Engl J Med. 2021;385(24):e83.
5. Chemaitelly H, Ayoub HH, AlMukdad S, Faust JS, Tang P, Coyle P, et al. Bivalent mRNA-1273.214 vaccine effectiveness against SARS-CoV-2 omicron XBB* infections. J Travel Med. 2023;30(5).
6. Altarawneh HN, Chemaitelly H, Hasan MR, Ayoub HH, Qassim S, AlMukdad S, et al. Protection against the Omicron Variant from Previous SARS-CoV-2 Infection. N Engl J Med. 2022;386(13):1288-90.
7. Chemaitelly H, Ayoub HH, Tang P, Coyle P, Yassine HM, Al Thani AA, et al. Long-term COVID-19 booster effectiveness by infection history and clinical vulnerability and immune imprinting: a retrospective population-based cohort study. Lancet Infect Dis. 2023;23(7):816-27.
8. Abu-Raddad LJ, Chemaitelly H, Ayoub HH, Al Kanaani Z, Al Khal A, Al Kuwari E, et al. Characterizing the Qatar advanced-phase SARS-CoV-2 epidemic. Sci Rep. 2021;11(1):6233.
9. Chemaitelly H, Bertollini R, Abu-Raddad LJ, National Study Group for Covid Epidemiology. Efficacy of Natural Immunity against SARS-CoV-2 Reinfection with the Beta Variant. N Engl J Med. 2021;385(27):2585-6.
10. Abu-Raddad LJ, Chemaitelly H, Ayoub HH, AlMukdad S, Yassine HM, Al-Khatib HA, et al. Effect of mRNA Vaccine Boosters against SARS-CoV-2 Omicron Infection in Qatar. N Engl J Med. 2022;386(19):1804-16.
11. Vogels C, Fauver J, Grubaugh N. Multiplexed RT-qPCR to screen for SARS-COV-2 B.1.1.7, B.1.351, and P.1 variants of concern V.3. dx.doi.org/10.17504/protocols.io.br9vm966. 2021(June 6, 2021).
12. Abu-Raddad LJ, Chemaitelly H, Butt AA, National Study Group for Covid Vaccination. Effectiveness of the BNT162b2 Covid-19 Vaccine against the B.1.1.7 and B.1.351 Variants. N Engl J Med. 2021;385(2):187-9.
13. Chemaitelly H, Yassine HM, Benslimane FM, Al Khatib HA, Tang P, Hasan MR, et al. mRNA-1273 COVID-19 vaccine effectiveness against the B.1.1.7 and B.1.351 variants and severe COVID-19 disease in Qatar. Nat Med. 2021;27(9):1614-21.
14. National Project of Surveillance for Variants of Concern and Viral Genome Sequencing. Qatar viral genome sequencing data. Data on randomly collected samples. https://www.gisaid.org/phylodynamics/global/nextstrain/ 2021 [Available from: https://www.gisaid.org/phylodynamics/global/nextstrain/.
15. Benslimane FM, Al Khatib HA, Al-Jamal O, Albatesh D, Boughattas S, Ahmed AA, et al. One Year of SARS-CoV-2: Genomic Characterization of COVID-19 Outbreak in Qatar. Front Cell Infect Microbiol. 2021;11:768883.
16. Hasan MR, Kalikiri MKR, Mirza F, Sundararaju S, Sharma A, Xaba T, et al. Real-Time SARS-CoV-2 Genotyping by High-Throughput Multiplex PCR Reveals the Epidemiology of the Variants of Concern in Qatar. Int J Infect Dis. 2021;112:52-4.
17. Saththasivam J, El-Malah SS, Gomez TA, Jabbar KA, Remanan R, Krishnankutty AK, et al. COVID-19 (SARS-CoV-2) outbreak monitoring using wastewater-based epidemiology in Qatar. Sci Total Environ. 2021;774:145608.
18. El-Malah SS, Saththasivam J, Jabbar KA, K KA, Gomez TA, Ahmed AA, et al. Application of human RNase P normalization for the realistic estimation of SARS-CoV-2 viral load in wastewater: A perspective from Qatar wastewater surveillance. Environ Technol Innov. 2022;27:102775.
19. Tang P, Hasan MR, Chemaitelly H, Yassine HM, Benslimane FM, Al Khatib HA, et al. BNT162b2 and mRNA-1273 COVID-19 vaccine effectiveness against the SARS-CoV-2 Delta variant in Qatar. Nat Med. 2021;27(12):2136-43.
20. Chemaitelly H, Ayoub HH, AlMukdad S, Coyle P, Tang P, Yassine HM, et al. Duration of mRNA vaccine protection against SARS-CoV-2 Omicron BA.1 and BA.2 subvariants in Qatar. Nat Commun. 2022;13(1):3082.
21. Qassim SH, Chemaitelly H, Ayoub HH, AlMukdad S, Tang P, Hasan MR, et al. Effects of BA.1/BA.2 subvariant, vaccination and prior infection on infectiousness of SARS-CoV-2 omicron infections. J Travel Med. 2022;29(6).

22. Altarawneh HN, Chemaitelly H, Ayoub HH, Hasan MR, Coyle P, Yassine HM, et al. Protective Effect of Previous SARS-CoV-2 Infection against Omicron BA.4 and BA.5 Subvariants. N Engl J Med. 2022;387(17):1620-2.
23. Chemaitelly H, Tang P, Coyle P, Yassine HM, Al-Khatib HA, Smatti MK, et al. Protection against Reinfection with the Omicron BA.2.75 Subvariant. N Engl J Med. 2023;388(7):665-7.
